# Self-Reported Uptake of Clinical Preventive Services by Vision Impairment Status

**DOI:** 10.1093/geroni/igaa057.695

**Published:** 2020-12-16

**Authors:** Lama Assi, Ahmed Shakarchi, Orla Sheehan, Nicholas Reed, Bonnielin Swenor

**Affiliations:** 1 Johns Hopkins Wilmer Eye Institute, Baltimore, Maryland, United States; 2 Johns Hopkins University School of Medicine, Baltimore, Maryland, United States; 3 Johns Hopkins University, Baltimore, Maryland, United States

## Abstract

Disease prevention is central to healthy aging. People with vision impairment are more likely than those without to report barriers to accessing health care and have unmet health care needs. We examined the association between functional vision impairment and preventive care uptake among adults aged 65 years and older in the 2016 and 2018 Behavioral Risk Factor Surveillance System (BRFSS) survey. The outcome of interest was being up-to-date with the recommended core clinical preventive services, as defined by Healthy People 2020: influenza and pneumococcal vaccination, and colorectal cancer screening for men, with the addition of breast cancer screening for women. Self-reported vision impairment was defined as blindness or serious difficulty seeing, even when wearing glasses. In models adjusted for sociodemographic characteristics (including age), access to care, and health/functional status, there was no difference in the odds of reporting being up-to-date with the recommended core preventive services among men with vision impairment compared to those without (odds ratio [OR]=0.90, 95% confidence interval [CI]=0.8-1.01); however, men with vision impairment were 0.82 times (95% CI=0.71-0.94) less likely than those without to report being up-to-date with colorectal cancer screening. Women with vision impairment were less likely than those without to report being up-to-date with the recommended core preventive services (OR=0.77, 95% CI=0.69-0.87); among the different services, the odds were lowest for reporting breast and colorectal cancer screening. These findings suggest that to achieve higher rates of preventive care uptake, especially cancer screening, older adults with vision impairment may be a special group to target.

